# Impact of Erythropoietin Production by Erythroblastic Island Macrophages on Homeostatic Murine Erythropoiesis

**DOI:** 10.3390/ijms21238930

**Published:** 2020-11-25

**Authors:** Genève Perron-Deshaies, Philippe St-Louis, Hugo Romero, Tatiana Scorza

**Affiliations:** 1 Département des Sciences Biologiques, Université du Québec à Montréal, Montreal, QC H3C 3P8, Canada; perron-deshaies.geneve@courrier.uqam.ca (G.P.-D.); st-louis.philippe.2@courrier.uqam.ca (P.S.-L.); hugo.romero.pro@gmail.com (H.R.); 2 CHU Sainte-Justine Research Centre, Montreal, QC H3T 1C5, Canada

**Keywords:** steady state erythropoiesis, macrophages, EPO, apoptosis, EBI

## Abstract

Erythropoietin (EPO) is an essential hormone for erythropoiesis, protecting differentiating erythroblasts against apoptosis. EPO has been largely studied in stress or pathological conditions but its regulatory role in steady state erythropoiesis has been less documented. Herein, we report production of EPO by bone marrow-derived macrophages (BMDM) in vitro, and its further enhancement in BMDM conditioned with media from apoptotic cells. Confocal microscopy confirmed EPO production in erythroblastic island (EBI)-associated macrophages, and analysis of mice depleted of EBI macrophages by clodronate liposomes revealed drops in EPO levels in bone marrow (BM) cell lysates, and decreased percentages of EPO-responsive erythroblasts in the BM. We hypothesize that EBI macrophages are an in-situ source of EPO and sustain basal erythropoiesis in part through its secretion. To study this hypothesis, mice were injected with clodronate liposomes and were supplied with exogenous EPO (1–10 IU/mouse) to evaluate potential rescue of the deficiency in erythroid cells. Our results show that at doses of 5 and 10 IU, EPO significantly rescues BM steady state erythropoiesis in mice deficient of macrophages. We propose existence of a mechanism by which EBI macrophages secrete EPO in response to apoptotic erythroblasts, which is in turn controlled by the numbers of erythroid precursors generated.

## 1. Introduction

The role of macrophages in the sustainment of erythropoiesis is an active field of study. Since the discovery of erythroblastic islands (EBI) by M. Bessis in 1958 [1], several functions have been described for central macrophages, including stimulation of erythroblastic proliferation, iron distribution and phagocytosis of extruded nuclei [2,3,4,5,6]. Macrophages have also been shown to promote erythroblastic survival, but the mechanism implicated in the process is poorly understood [6].

A study published in 1988 reported erythropoietin (EPO) expression by bone marrow (BM) macrophages [7], but its pertinence in erythropoiesis was not further studied. More recently, a paper published by Luo et al. (2016) reported secretion of EPO by proximal macrophages when stimulated by apoptotic cell-derived sphingosine-1-phosphate (S1P). This EPO production had an autocrine effect on the macrophage, stimulating expression of EPO-receptor (EPO-R) and inducing an M2-like phenotype in these cells, which enhanced their phagocytic properties (activation) and production of anti-inflammatory cytokines [8].

Erythropoiesis is regulated in part by the apoptosis of erythroblasts. In homeostatic conditions, progenitors are produced in excess, and in situ EPO concentrations allow for the survival of only a few of these cells, the remaining undergoing apoptosis. Based on this information, we hypothesized that EBI central macrophages are an in-situ source of EPO and that its production is regulated by surrounding apoptotic cells.

To study this hypothesis, bone marrow derived macrophages (BMDM) were conditioned with apoptotic cell media and were assessed for EPO production in vitro. EBI were isolated from mice for detection of intracellular EPO by confocal imagery (immunofluorescence, IF). Finally, the ability of physiological doses of recombinant human EPO (rHuEPO) to restore the deficient erythropoietic response in clodronate-treated mice was investigated.

## 2. Results

### 2.1. BMDM Conditioned with Apoptotic Cell-Derived Media Have Improved Ability to Sustain Erythropoiesis In Vitro

**Figure 1:**
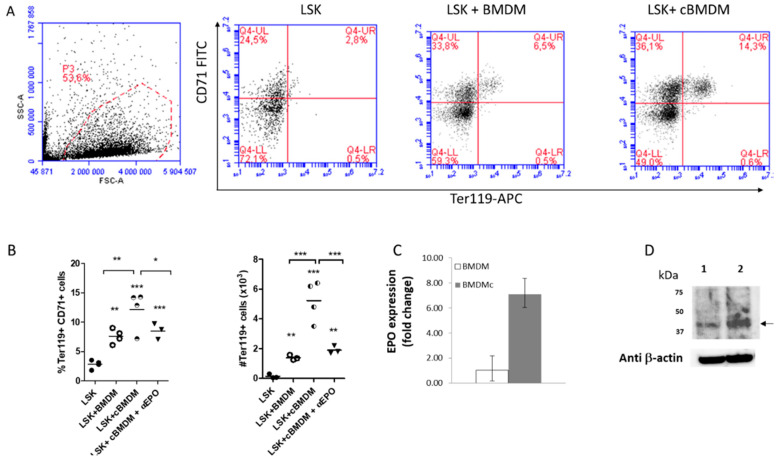
Bone marrow-derived macrophages produce erythropoietin (EPO) and stimulate erythroid cells differentiation in vitro. (**A**) Lineage^-^cKit^+^Sca1^+^ (LSK) cells were cultured for 5 days in an erythropoietic media without exogenous EPO, alone or in the presence of BMDM or BMDM conditioned with media from apoptotic splenocytes (cBMDM). CD71/Ter119 expression was analyzed by flow cytometry. (**B**) Data from three independent experiments show higher percentages and numbers of Ter119^+^ cells in BMDM-LSK co-cultures, an effect significantly enhanced when cBMDM were used, and partially abrogated by an EPO neutralizing antibody. (**C**) Enhanced expression of EPO-specific mRNA as measured in cBMDM when compared to BMDM in two independent qRT-PCR assays. (**D**) Western blot analysis of EPO production from control BMDMs (1) and cBMDMs (2), densitometry analysis indicating two-fold EPO expression in the latter. Β-actin was used as a reference gene. *** *p* < 0.001, ** *p* < 0.01, * *p* < 0.05.

Interaction of murine peritoneal macrophages with apoptotic cells induces EPO secretion by macrophages, which in turn stimulates phagocytosis and elimination of apoptotic cells [8]. Considering that elevated numbers of bone marrow and spleen Ter119^+^ cells enter apoptosis in steady stage conditions (Supplementary Figure S1A–F, we investigated whether conditioning of BMDM with supernatants from ~50% apoptotic cells (Supplementary Figure S1F) rendered these cells more apt to support erythroid differentiation. For this, Lineage^−^Sca1^+^cKit^+^ stem cells (LSK) were enriched by negative selection and cultured in media without the addition of exogenous EPO in the absence or presence of control or conditioned BMDM at a ratio of 4:1 ratio, respectively, for five days. Co-culture of LSK with BMDM resulted in the appearance of Ter119^+^CD71^+^ cells in the culture (Figure 1A,B), the effect being enhanced when BMDM were preconditioned with media from apoptotic splenocytes for 16 h, and significantly abrogated by an EPO neutralizing antibody (Figure 1B). In accordance with these results, EPO protein was confirmed in lysates from BMDM and its expression was increased two-fold in conditioned BMDM (Figure 1C). The fact that an EPO specific ELISA was not sensitive enough to detect EPO in BMDM-derived supernatants suggest that this hormone is secreted at relatively low levels (less than 1 pg/mL; data not shown). These results suggest that BMDM-derived EPO stimulates the differentiation of LSK into Ter119^+^CD71^+^ cells under the in vitro conditions tested, and that the effect is further enhanced in BMDM preconditioned with apoptotic cell media.

### 2.2. Detection of EPO in Ex-Vivo EBI Macrophages by IF and Confocal Imagery

**Figure 2:**
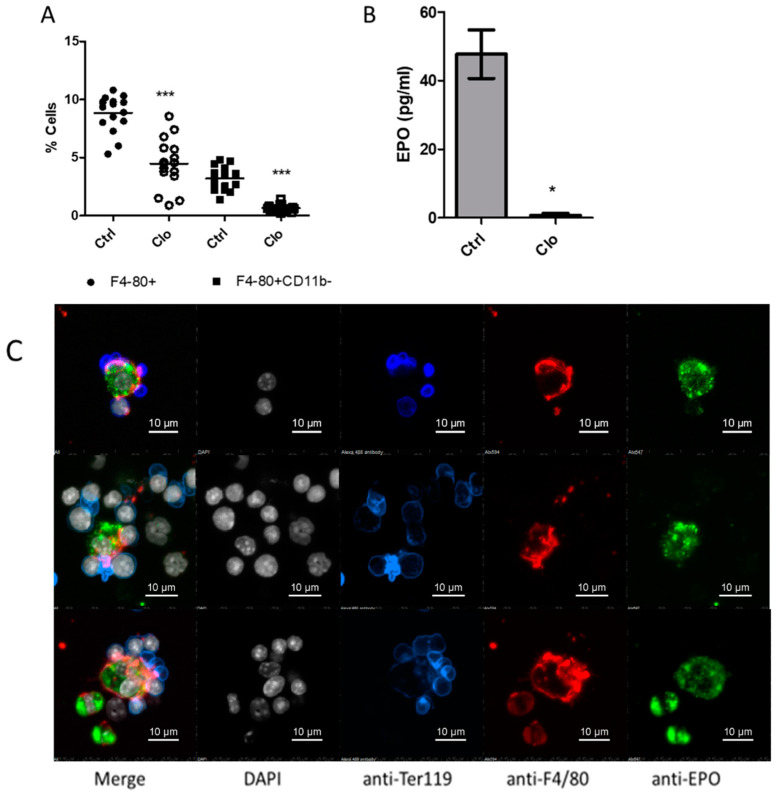
Analysis of EPO concentrations in bone marrow cell lysates from mice depleted of macrophages, and confirmation of EPO expression in erythroblastic island (EBI)-associated macrophages. (**A**) Analysis of F4/80^+^ and F4/80^+^CD11b^−^ cells in the bone marrow (BM) 7 days post-administration of clodronate or PBS-containing liposomes in mice. (**B**) Twenty million BM cells were lysed in RIPA buffer and EPO concentration was assayed by ELISA. (**C**) Total femur cells were flushed and treated with Brefeldin A for 4 hours. Adherent cells were stained with anti-Ter119 Alexa 488 (blue) and anti-F4/80 Alexa 594 (red). Cells were fixed and permeabilized, then stained with anti-EPO Alexa 647 (green) and DAPI (grey). EBI macrophages were identified as F4/80^+^ cells surrounded by Ter119^+^ cells. *** *p* < 0.001, * *p* < 0.05.

Having confirmed in vitro production of EPO by BMDM, we assessed the impact of clodronate liposomes on EPO levels in the BM, five days following their administration. Clodronate-treated mice had significantly lower percentages of F4/80^+^ cells and F4/80^+^CD11b^−^ cells (a phenotype associated with EBI macrophages) in femoral BM (Figure 2A, *p* < 0.001). EPO content in supernatants from BM cell lysates was assessed by ELISA, and was found to be significantly lower in clodronate treated mice (Figure 2B, *p* < 0.05), suggesting that macrophages are an important source of EPO in the BM. The specific contribution of EBI macrophages as EPO producers was confirmed by confocal microscopy (Figure 2C), with EPO being detected in macrophages surrounded by erythroblasts. Altogether, these results confirm that EBI-associated macrophages represent an in-situ source of EPO for differentiating erythroblasts.

### 2.3. Impact of Macrophage Depletion with Clodronate Liposomes on Murine Erythropoiesis

**Figure 3:**
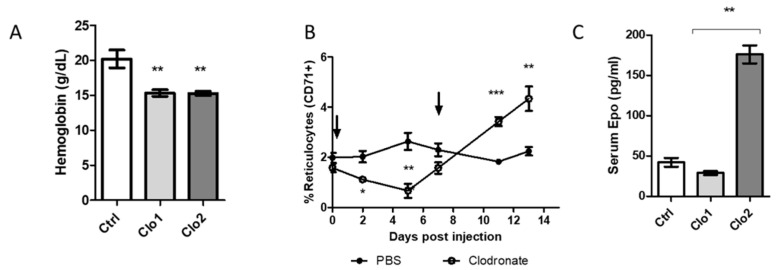
Impact of clodronate liposomes on blood hemoglobin, reticulocytes and serum levels of EPO. Macrophages were ablated by a single (1 week length) or two (2 weeks length) injections of clodronate liposomes. (**A**) Hemoglobin levels in peripheral blood were measured with the Drabkin method. (**B**) Percentages of circulating reticulocytes were quantified in blood with an anti-CD71 antibody. (**C**) Serum EPO concentrations were determined by ELISA. *** *p* < 0.001, ** *p* < 0.01, * *p* < 0.05.

Several studies have reported impaired steady state erythropoiesis in mice deprived of macrophages by clodronate liposomes or genetic ablation [3,9]. We compared the impact of one or two injections of clodronate liposomes in mice on hemoglobin and reticulocyte concentrations in peripheral blood. Clodronate was administered once per week, and mice were sacrificed 7 or 14 days later. Blood analysis confirmed comparable drops in hemoglobin concentrations in clodronate-treated mice, when compared to mice receiving PBS-containing liposomes (Figure 3A, *p* < 0.05). Analysis by flow cytometry revealed significant drops in blood CD71^+^ cells at days 2 and 5 post administration of clodronate liposomes, confirming impaired steady state erythropoiesis (Figure 3B). From day 7 on, the percentages of reticulocytes gradually increased, becoming significantly higher in mice that received a second injection of clodronate liposomes at days 12 and 14 when compared to control mice (Figure 3B). As expected, the enhanced reticulocytosis was accompanied by ~4-fold increases in serum EPO levels at day 14 (Figure 3C, *p* < 0.01), suggesting induction of stress erythropoiesis.

**Figure 4:**
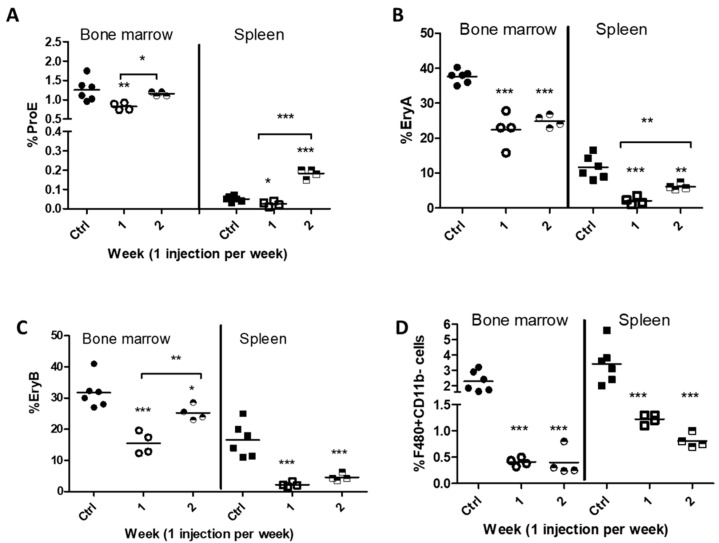
Effect of macrophage depletion on bone marrow and splenic erythroid cells. (**A**) Percentages of proerythroblasts (ProE) in the BM and spleen one (1) or two (2) weeks following administration of clodronate liposomes with respect to control mice that received PBS-containing liposomes. (**B**) Percentages of polychromatic (EryA) and (**C**) orthochromatic (EryB) erythroblasts within the Ter119high population in the BM and spleen. (**D**) Percentages of F4/80^+^CD11b^−^ cells in the BM and spleen. *** *p* < 0.001, ** *p* < 0.01, * *p* < 0.05.

Detailed analysis of erythroid cells was then done with BM and splenic cells, following co-staining with anti-Ter119 and anti-CD71 antibodies and discrimination of Ter119^high^ cells by size and expression of CD71, as initially reported by Liu et al. [10] (see gating strategies in Figures S3 and S4). Proerythroblasts (ProE), distinguished as cells with moderate expression of Ter119 and high CD71 expression, dropped significantly in the BM and spleen 7 days after a first clodronate injection (Figure 4A), their percentages being restored at day 14 in the BM and significantly increased in the spleen (Figure 4A). Discrimination of basophilic (EryA) as Ter119^high^CD71^+^FSC^high^ cells confirmed their reduction in the BM and spleen a week after clodronate administration (Figure 4B). A comparable effect was measured in polychromatic (EryB) erythroblasts (Ter119^high^CD71^+^FSC^low^ cells), which dropped significantly at day 7 post injection (Figure 4C). Our data indicate complete restoration of ProE and partial restoration of EryA and EryB cells in the BM when serum EPO levels rise, despite persisting low numbers of F4/80^+^CD11b^−^ cells (Figure 4D).

### 2.4. Restoration of Erythropoiesis by Exogenous EPO in Depleted Mice

The data so far presented is indicative of impaired steady state erythropoiesis in mice ablated of macrophages for one week, and induction of a stress response in the spleen if macrophage ablation is sustained for two weeks, characterized by increased percentages of ProE cells and accompanied by a 4-fold increase in blood EPO levels and enhanced reticulocytosis.

Our next objective aimed at assessing the ability of exogenous EPO to restore the deficient erythropoietic response caused by short term depletion of EBI (F4/80^+^CD11b^−^) macrophages. For this, three different doses of EPO were administered to mice at day 1 and day 2 following a single clodronate liposome injection. All three doses were physiologically relevant and significantly lower than those used to induce a potent stress response.

**Figure 5:**
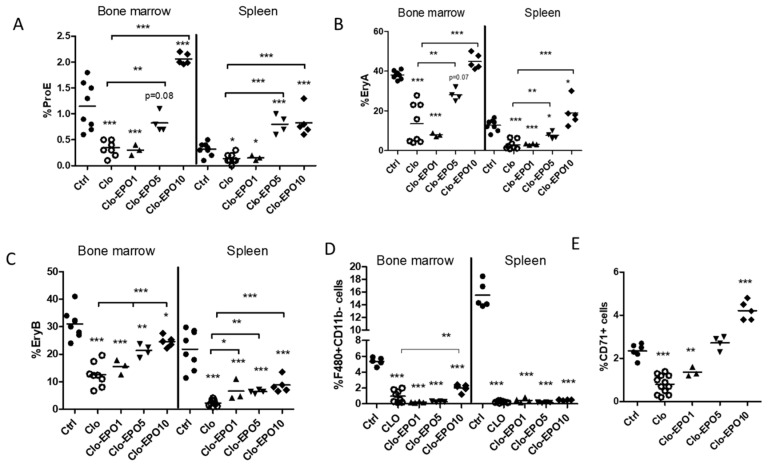
Effect of exogenous EPO on bone marrow and splenic erythroblasts populations from mice treated with clodronate liposomes. Proportions of (**A**) ProE, (**B**) EryA, and (**C**) EryB in the bone marrow and the spleen from control (Ctrl), clodronate-treated (Clo) mice, and clodronate-treated mice receiving rHuEPO at 1 IU (Clo-EPO1), 5 IU (Clo-EOI5) or 10 IU (Clo-EPO10) doses. (**D**) Percentages of F4-80+CD11b− cells in the BM and spleen. (**E**) Reticulocytes (CD71+ cells) in blood at day 4 post-treatment. BALB/c mice were injected with a single dose of clodronate liposomes and treated with rHuEPO 24 and 48 h after. Mice were sacrificed at day 4. *** *p* < 0.001, ** *p* < 0.01, * *p* < 0.05.

As shown in Figure 4 and Figure 5, administration of clodronate liposomes caused significant drops in the ProE, EryA and EryB populations, which are stages expressing the EPO-R, ProE being the most responsive to EPO [10]. At 1 IU, EPO did not reestablish ProE in the BM or spleen (Figure 5A–C), but a 5 IU dose efficiently restored ProE in the BM, and enhanced their production in the spleen of clodronate-treated mice when compared to untreated controls. The 10 IU EPO dose was stimulatory in both organs when compared to naïve mice (Figure 5A). A similar restoration was measured for EryA in the BM and spleen when compared to clodronate-treated mice. With respect to EryB, although their proportion gradually increased in the BM and spleen in correlation with EPO concentrations, complete restoration was not observed. It is important to mention that F4/80^+^CD11b^−^ cells remained significantly low for all conditions tested (Figure 5D). In agreement with these data, reticulocytosis was restored following administration of 5 IU EPO in clodronate-treated mice and enhanced above control levels by the largest dose tested.

## 3. Discussion

EPO has been extensively studied in the past years in the context of anemia therapies or erythrocytosis [11,12,13,14,15,16,17,18]. The role of EPO in stress erythropoiesis has been well documented, with renal peritubular cells being the major source for this hormone [19]. While it is accepted that EPO is necessary to maintain homeostatic erythropoiesis [4,20,21,22], its source under basal conditions has been mainly attributed to the kidney.

Basal erythropoiesis is regulated by the rate of apoptosis of erythroblasts and the elimination of senescent RBC by red pulp macrophages. In steady state conditions, most erythroid progenitors die. During hypoxic or anemic stress, the lack of oxygen is detected by the kidney and EPO production is activated by hypoxia-inducible transcription factors (HIF). The resulting heightened concentrations of EPO allow survival of more progenitors, quickly increasing the production of erythrocytes [4,23].

A study published in 2016 revealed a link between macrophages, apoptosis, and EPO production. Luo et al. demonstrated that sphingosine 1 phosphate (S1P) from apoptotic cells induces EPO production in proximal peritoneal macrophages, which in turn acts in an autocrine manner to promote uptake and elimination of apoptotic cells [8]. These actions are valuable within erythropoietic niches in steady state conditions, first because of the relative elevated rate of apoptotic erythroblasts, and second, because of the important action that EPO has on ProE, EryA and EryB, which are the stages entering apoptosis [10]. It is therefore expected that discrete amounts of EPO, released by central macrophages in response to erythroblast apoptosis, may affect EPO-R expressing erythroblasts in close contact, thus providing survival signals.

To investigate this possibility, we first evaluated the pro-erythropoietic effects of BMDM. Our results indicated that on their own, BMDM sustain differentiation of LSK cells into Ter119^+^ cells, and that this is further enhanced in BMDM preconditioned with media from apoptotic cells. The latter produced more EPO, as confirmed by qRT-PCR and Western Blot. However, unlike the data obtained with peritoneal macrophages by Luo et al. [8], the amounts of EPO released by BMDM were undetectable by ELISA, suggesting that BMDM produce small amounts of this hormone. However, the relative contribution for BMDM-derived EPO on erythropoiesis was confirmed with an EPO neutralizing antibody, which abrogated, although not completely, the pro-erythropoietic action of BMDM conditioned with apoptotic media. An interesting study by Rhodes et al. [24] with splenic macrophages and erythroid cells demonstrated the importance of adherence between macrophages and erythroblasts on erythroid differentiation. Although addition of EPO was shown to be relevant for the survival of erythroblasts, the presence of macrophages resulted in three-fold increases in the numbers of erythroblasts in culture, demonstrating that factors such as cell adhesion molecules also contribute to these effects.

Having collected evidence linking macrophage-derived EPO with erythropoiesis in vitro, we then assessed the impact of macrophage ablation with clodronate-liposomes on steady state BM erythropoiesis. Our first observation was that EPO levels were significantly lower in BM-derived cell lysates from mice ablated of F4/80^+^CD11b^−^ cells for seven days, which suggests that clodronate liposomes eliminate possible sources of EPO in the BM. Considering that no major effects were measured in plasmatic EPO concentrations, the impact of clodronate-liposomes in BM-derived EPO seems independent of systemic down regulation of EPO levels. Second, confocal imagery confirmed intracellular EPO in F4/80^+^ macrophages in closed contact with Ter119^+^ cells in the BM, suggesting that ablation of macrophages maybe a cause of decreased EPO levels in the BM.

In vivo data from clodronate-treated mice showed a common trend in the BM and spleen: early, EPO-R expressing erythroblasts (ProE, EryA and EryB) are the most affected by macrophage depletion. Elimination of macrophages for one week resulted in impaired BM erythropoiesis, as shown by the drops in reticulocyte and hemoglobin concentrations in peripheral blood. If sustained for two weeks, macrophage ablation led to increased levels of EPO in sera as well as enhanced reticulocytosis, indicating induction of a stress response. The spleen was probably involved in the latter, as reflected by rises in ProE and relative restoration of EryA in this organ. The drops in early erythroblastic populations, and the ensuing stress response, may thus be in part attributed to deficient EPO in erythropoietic niches.

A second set of in vivo experiments were conducted to evaluate the extent to which early administration of EPO rescued the deficient erythropoiesis in clodronate-treated mice. For this, clodronate-depleted mice were injected with physiologically relevant doses of rHuEPO (1 IU, 5 IU and 10 IU per mice). Homeostatic EPO levels are around 0.010 IU/mL of blood. During severe stress (like anemia or hypoxia) kidney peritubular cells heighten EPO concentrations to as high as 10 IU/mL. [16,20]. By injecting a maximum of 10 IU per mouse, we ensured that our treatment would not go beyond physiological EPO levels.

The BM and spleen showed complete restoration of ProE and EryA populations after treatment with 10 IU rHuEPO whereas EryB were significantly restored. A probable explanation for incomplete recovery in EryB may be the time point at which mice were sacrificed for analysis. We did not assess the numbers of Burst forming units-erythroid (BFU-E) or Colony forming units-erythroid cells (CFU-E). About 48 h are presumed to be required for a CFU-E to differentiate into an EryB [25,26]. The total length of the EPO treatment being of 72 h, new EPO-stimulated CFU-E might be short on time to get to that stage, especially in conditions deprived of macrophages.

An additional interesting point concerns EBIs on their own. We did not assess the extent to which EBI are restored in EPO-treated mice deprived of macrophages, which is an interesting issue that may involve cells other than macrophages or special adaptations to poor macrophage conditions.

The fact that soluble factors derived from apoptotic cells enhance the ability of BMDM to support erythropoiesis (also confirmed with S1P-conditioned BMDM, data not shown) allows for proposing the existence of a fine-tuned mechanism, triggered and sustained by an excessive production of erythroblasts in vivo. The fact that significantly lower rates of apoptotic Ter119+CD71+ cells were measured in clodronate-treated mice, which generate significantly lower numbers of erythroblasts, reinforces the important association between erythroblast numbers and apoptosis (Supplementary Figure S2). In this respect, EPO is not the sole regulator of erythroblast apoptosis. An additional element concerns the interactions between the cell-death receptor Fas (CD95) and its ligand (Fas-L, CD95L, CD178). Activation of Fas trigger apoptosis of cells expressed through the caspase-3 apoptotic complex. [27] This complex plays an important role in terminal erythroid differentiation and EPO prevents apoptosis downstream of the caspase activation [27,28]. Since these two proteins are expressed by early erythroblasts [10] a fast and massive drop in EryA and EryB numbers, would decrease the density of the erythroid cell population, reducing Fas-FasL interaction, and thus, decreasing apoptosis.

Certain aspects of our study, especially those concerning the structure of EBI in macrophage-deficient conditions, would be interesting to further explore. The differences between splenic and BM EBI, which are possibly linked to different signals operating during the murine stress response in the spleen, are also an interesting subject that has been extensively explored and has been discussed in excellent reviews [2,23].

In conclusion, we have shown that EBI macrophages are an in-situ source of EPO for erythroblasts, and that this EPO contributes to the regulation of homeostatic erythropoiesis. We propose the existence of a mechanism by which EBI macrophages secrete EPO in response to apoptotic erythroblasts, which is in turn controlled by the numbers of erythroid precursors generated.

## 4. Materials and Methods

### 4.1. In Vitro Experiments

#### 4.1.1. Generation of BMDM

BMDMs were generated from non-adherent bone marrow cell suspension from BALB/c mice. Bone marrow was flushed from the femur and tibia by centrifugation 5 min at 3000× *g* in a microcentrifuge, pooled and cultured for 4 h to eliminate differentiated macrophages. Non-adherent cells were counted and plated in a 96 well plate at a density of 0.375 × 10^6^ cells per mL in 0.2 mL of DMEM supplemented with 10% FBS, 100 IU/mL penicillin/streptomycin and 20% L929-enriched medium. Cells were cultured for 7 days with a change of media on day 3 to generate a pure BMDM population.

#### 4.1.2. Generation of Apoptotic Splenocyte, Apoptotic Media and Conditioning of BMDMs

Whole splenocytes were harvested from spleen of BALB/c mice by mechanical disruption. Whole spleen was plated in tissue culture treated flask (Sarstedt, Newton, MA, USA) at a density of 4 × 10^6^ cells/mL for 4 h and non-adherent cells were transferred into a new vessel. Natural apoptosis was induced by culture for 48 h and verified by Annexin V/7AAD apoptosis detection kit (Biolegend, San Diego, CA, USA). For conditioning with apoptotic media, the supernatant was filtered using a 0.22-micron filter and used as is. For conditioning with apoptotic splenocytes, cells were counted and put in co-culture with BMDMs at a ratio of 5 apoptotic cells per BMDM.

#### 4.1.3. Hematopoietic Stem Cells Co-Culture with BMDM

Differentiated BMDMs were conditioned with apoptotic media overnight. The following day, bone marrow from a BALB/c mouse was harvested and hematopoietic stem cells were enriched by negative selection (Stemcell technologies, Vancouver, BC, Canada). Elimination of Ter119^+^ cells was confirmed by FC. BMDMs were washed three times with PBS and LSKs were added to the wells at a ratio of 4 LSK cells per BMDM in an erythropoietic media modified from Shuga et al. (2007) [29], consisting of IMDM (Wisent, Montreal, QC, Canada) supplemented with FBS 10%, P/S 50U/mL (Sigma Aldrich, Oakville, ON, Canada), holotransferin 200 ug/mL (SigmaAldrich,), insulin 10ug/mL (Sigma Aldrich), β-mercaptoethanol 10^−4^ M (Sigma Aldrich), dexamethasone 10^−5^ M (Vetoquinol, Lavaltrie, Canada), SCF 10ng/mL (R&D Systems, Minneapolis, MN, USA) and IGF-1 (R&D Systems). After 5 days of co-culture, non-adherent cells were analyzed by FC for erythroblastic engagement using anti-CD71 FITC, anti-Ter119 APC and 7AAD (Biolegend). EPO neutralization used the rat IgG2a monoclonal anti-EPO antibody, clone 148,436 (Invitrogen) at a 0.75 ug/mL concentration during the five days of culture.

#### 4.1.4. Analysis of EPO Transcript by RT-qPCR

BMDMs were conditioned with apoptotic media or DMEM culture media for 4 h. Afterwards, total RNA was obtained using the Aurum Total RNA mini kit (Bio-Rad, Missisauga, ON, Canada) and 1 ug was turned into cDNA using iScript reverse transcription supermix for RT-qPCR (Bio-Rad). qPCR was performed with 50 ng of cDNA in an ABI7300 system (Applied Biosystems, Foster City, CA, USA) with 30 s of activation at 95 °C followed by a 40 cycles 2 step program of 15 s denaturation at 95 °C and 60 s of annealing. SsoAdvanced universal SYBR Green supermix was used as a probe with primePCR assay primers for EPO (Bio-Rad #10025636) and GADPH (Bio-Rad #10025636). A meltcurve analysis was done afterward to ensure single product amplification. Expression of EPO was normalized to GADPH and analysed using 7300 system software (Applied Biosystems).

#### 4.1.5. EPO Detection by Western Blot

BMDMs were conditioned overnight with apoptotic cells or DMEM culture media. The next day, BMDMs were washed three times with PBS and were incubated with Brefeldin A (Biolegend) for 4 h. Afterwards, cells were lysed with RIPA buffer (NaCl 150 mM, Tris 50 mM, NP40 1%, sodium deoxycholate 0.5%, SDS 0.1%). Protein concentration was determined with DC protein assay (Bio-Rad) and 10 µg of total protein was added per wells for SDS-PAGE. Transfer was performed with transblot turbo (Bio-Rad) using manufacturer standard semi-dry transfer protocol onto PVDF membrane (Millipore, Oakville, Canada). Membrane was blocked overnight with 3% BSA in TBS-T and probed with 1:1000 anti-EPO (Santa Cruz, Dallas, TX, USA, cat#SC-5290) overnight at 4 °C. After revelation, the membrane was stripped, blocked, and probed with 1:50,000 anti-beta actin (Sigma Aldrich) for 1 h at room temperature. Femto super signal ECL (Pierce, Waltham, MA, USA) was used as substrate and revelation was captured using the Chemidoc MP imaging system (Bio-Rad). Densitometry analysis were performed using image lab software (Bio-Rad) and EPO expression was normalized to beta-actin.

### 4.2. In Vivo Assays

#### 4.2.1. Biological Model

For all in vivo experiments, 4 to 6-week-old female BALB/c mice were obtained from Charles River Laboratories (Canada). All protocols using animals were approved by the Animal Protection Institutional Comity (CIPA) and followed the Canadian Council on Animal Care (CCAC) guidelines. (Protocols 01518-944, 1 May 2018 and 0519-944, 1 May 2019).

#### 4.2.2. Short and Long-Term Macrophage Depletion

Macrophage depletion was induced by intravenous (iv) injection of 150 μL clodronate (20 mg/mL) liposomes (Clophosome-A, FormuMax, Sunnyvale, CA, USA). Control mice received PBS-containing anionic liposomes prepared by the same company. Two experimental groups were studied: 1-week (group 1, *n* = 10) and 2-weeks (group 2, *n* = 4) depletion length. Liposomes were injected on day 0. Mice from the first group were sacrificed on day 7. Group 2 mice received a second clodronate injection on day 7 and were sacrificed on day 14.

#### 4.2.3. Determination of EPO Concentration in Bone Marrow from Clodronate Treated Mice by ELISA

Whole bone marrow was recovered from naïve or clodronate treated BALB/c mice. Forty million (4 × 10^6^) BM cells were lysed in 200 µL RIPA buffer and EPO concentration was determined by ELISA (Biolegend, cat#442707).

#### 4.2.4. Restoration of Macrophage-Depleted Mice Erythropoiesis by EPO

The restoration of the erythropoiesis in macrophage depleted mice was studied by supplying exogenous EPO (R&D systems, cat#287-TC-500). Animals received an intravenous (iv) injection of clodronate or PBS-containing liposomes on day 0, followed by subcutaneous (sc) injections of recombinant human EPO (rhuEPO) or PBS on days 1 and 2. Doses of EPO between 50 and 500 IU/Kg body weight, corresponding to 1 IU, 5 IU and 10 IU per mice, were administered, diluted in PBS. Four to five mice were used for each dose, with equal numbers of naïve, EPO-treated, and clodronate-treated animals. Animals were sacrificed on day 4.

#### 4.2.5. Flow Cytometry

BM was extracted from the femur and tibia by centrifugation for 5 min at 6500× *g*. Spleen cells were prepared by gentle crushing in RPMI. Cells were counted and 5 × 10^5^ cells were stained in PBS. For analysis of the macrophage population, the following antibodies were used: anti-CD11b (clone M1/70, Biolegend, cat#101206) conjugated with fluorescein isothiocyanate (FITC), anti-F4/80 (clone BM8, StemCell Technologies, Vancouver, Canada, cat#60027PE) conjugated with phycoerythrin (PE), 7AAD (Biolegend, cat#420404). For analysis of the erythroblastic population and of its apoptosis, anti-CD71 FITC (clone R17217, Biolegend, cat#113806), anti-Ter119 PE (clone TER119, Biolegend, cat#116208), 7AAD and Annexin V (Biolegend, cat#640920 conjugated with Allophycocyanin (APC) were used. Cells were stained on ice and protected from light for 1 h. Data acquisition was made with a BD Accuri C6 (BD Biosciences, Franklin Lake, NJ, USA). A gate excluding debris was made with a FSC-SSC dot plot, and 10,000 events inside that gate were collected from each sample. Data was analyzed with the BD Accuri C6 Software. Gating strategies are available in the Supplementary Data (Figures S1 and S2).

### 4.3. Detection of EBI Macrophage Intracellular EPO by IF and Confocal Imagery

BM cells were extracted from naïve BALB/c mice aged 4–8 weeks. Briefly, femurs were flushed with 3 mL of IMDM through 20 G needles, washed and collected by sedimentation in RPMI+ 30% FBS. Cells were seeded on an 8-well slide (Ibidi, Gräfelfing, Germany, cat#80826) and treated with Brefeldin A for 4 h. Non-adherent cells were removed, and the remaining cells were stained with anti-F4/80 Alexa fluor 596 (Clone BM8, Biolegend, cat#123140) and anti-Ter119 Alexa fluor 488 (clone TER119, StemCell Technologies, cat#60033AD). Cells were fixed and permeabilized with the RnD systems Fix-Perm kit (cat#FC012-100) and stained for intracellular EPO with anti-EPO Alexa fluor 647 (clone B-4, Santa Cruz, cat#SC-5290-AF647). Finally, cells were stained with DAPI (Santa Cruz) and analyzed by confocal microscopy using a Nikon A1R plus confocal microscope.

### 4.4. Statistical Analysis

All except one statistical analysis were performed with R version 3.6.2. Proportions of populations were analyzed with generalized linear models with a quasibinomial family, which best suits the binomial distribution of the data. Cell counts were analyzed with a generalized linear model with a quasipoisson family, which best suits the Poisson distribution of count data. When applicable, two models were used: (1) comparison of every treatment against the control group and (2) comparison of every treatment against the clodronate-depleted group. Hemoglobin was analyzed with a linear model. EPO in the serum and lysates was analyzed with GraphPad Prism 5 using a one-way ANOVA.

## Supplementary Materials

The following are available online at https://www.mdpi.com/1422-0067/21/23/8930/s1, Figure S1: Strategy to evaluate apoptotic cells in the bone marrow and spleen by flow cytometry; Figure S2: Assessment of total Ter119+ CD71+ cells and apoptosis levels in control mice and in mice treated with clodronate liposomes; Figure S3: Gating strategies for exclusion of debris and dead cells; Figure S4: Gating and staining strategy for quantification of erythroid cells.

## Abbreviations

EPO	Erythropoietin
EBI	Erythroblastic island
BM	Bone marrow
BMDM	Bone marrow derived macrophages
EPO-R	Erythropoietin-receptor
FC	Flow cytometry
IF	Immunofluorescence
WB	Western Blot
ELISA	Enzyme-linked immunosorbent assay
qrtPCR	Quantitative reverse-transcriptase polymerase chain-reaction
rHuEPO	Recombinant human erythropoietin
S1P	Sphingosine-1-phosphate
IV	Intravenous
SC	Subcutaneous
BMDM	Bone marrow-derived macrophage
SDS-PAGE	Sodium Dodecyl Sulfate – Polyacrylamide gel electrophoresis
