# Supplementary material for: Impact of Erythropoietin Production by Erythroblastic Island Macrophages on Homeostatic Murine Erythropoiesis
Source: Int J Mol Sci. 2020 Nov 25;21(23):8930. doi: 10.3390/ijms21238930 (PMC7728051; doi:10.3390/ijms21238930)
Supplement: Supplementary file 1 [file ijms-21-08930-s001.pdf]

International Journal of *Molecular Sciences*

Supplementary

Impact of Erythropoietin Production by Erythroblastic Island Macrophages on Homeostatic Murine Erythropoiesis

Genève Perron-Deshaies [1], Philippe St-Louis [1], Hugo Romero [1,2] and Tatiana Scorza [1,*]

[1] Département des Sciences Biologiques, Université du Québec à Montréal, Montréal H3C 3P8, Canada; perron-deshaies.geneve@courrier.uqam.ca (G.P.-D.); st-louis.philippe.2@courrier.uqam.ca (P.S.-L.); hugo.romero.pro@gmail.com (H.R.)

[2] CHU Sainte-Justine Research Centre, Montreal H3T 1C5, Canada

* Correspondence: scorza.tatiana@uqam.ca; Tel.: +1-514-9873000 (ext. 1918)

Received: 1 November 2020; Accepted: 23 November 2020; Published: date

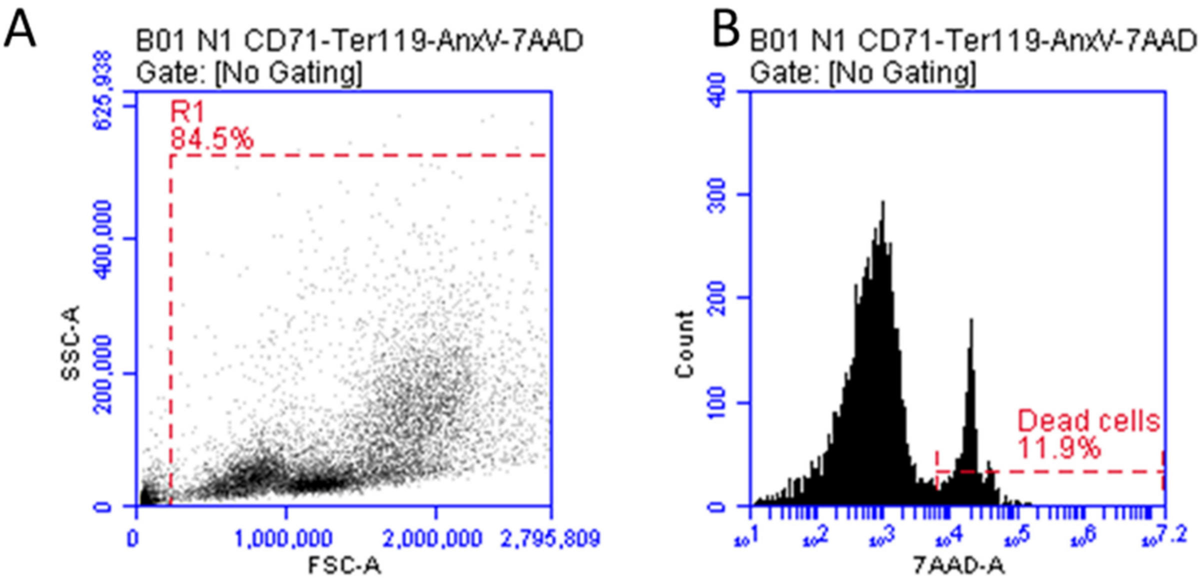

Figure 1. Gating strategies for the exclusion of debris and dead cells. (**A**) A gate was created broadly to include cells but exclude debris. For each sample, 10 000 events were collected inside this gate. (**B**) 7AAD+ cells were selected on a histogram plot for exclusion in later analysis.

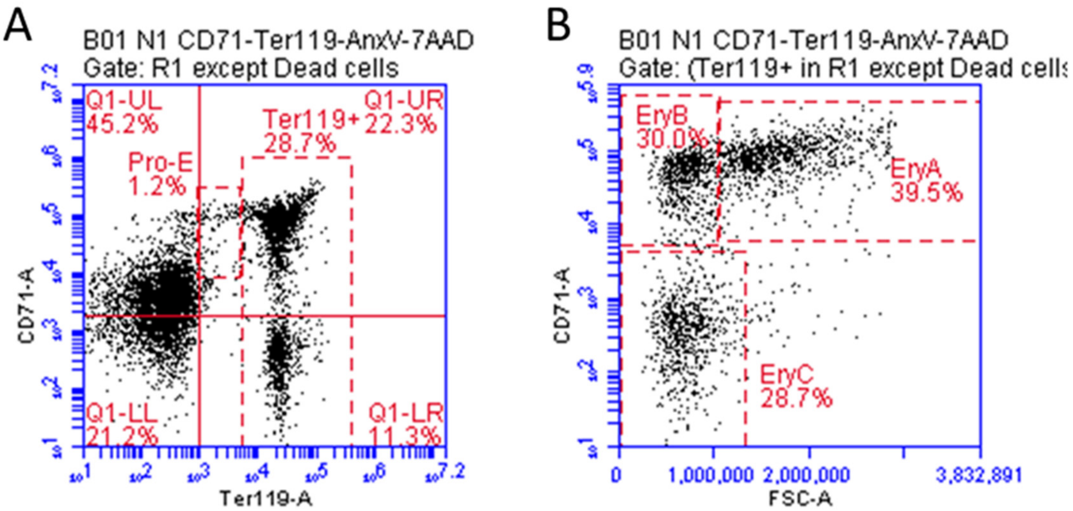

Figure 2. Gating strategies for identification of ProE, EryA, EryB and EryC. (**A**) Live cells were analyzed by expression of Ter119 and CD71. CD71^{high}Ter119^{med} cells were identified as proerythroblasts (ProE). A gate was created to select all Ter119^{high} cells. (**B**) Ter119^{high} cells were analyzed by size (FSC) and expression of CD71. EryA were identified as CD71^{high}FSC^{high}, EryB as CD71^{high}FSC^{low} and EryC as CD71^{low}FSC^{low}.

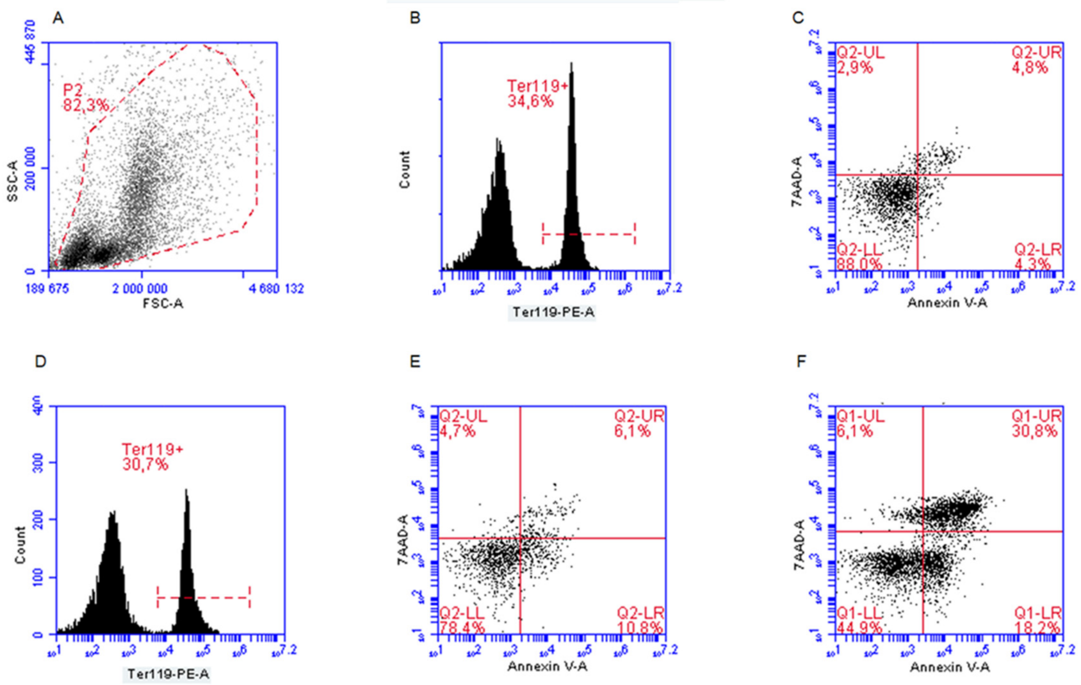

Figure 3. Strategy to evaluate apoptotic cells in the bone marrow and spleen by flow cytometry. Bone marrow and splenic cells were stained with anti Ter119 antibody, Annexin V and 7AAD. (**A**) Gate to exclude debris. (**B**) Selection of Ter119+ cells in the BM and (**C**) assessment of Annexin V+ and Annexin V+7AAD+ cells within BM Ter119+ cells. (**D**) Selection of Ter119+ in the spleen and (**E**) analysis the percentages of annexin V+ and annexinV+7AAD+ cells within Ter119+ cells in the spleen. (**F**) Splenic apoptotic erythroblasts following 48 hours in culture; supernatants from these cells were used to condition BMDM prior to their co-culture with LSK cells for evaluation of pro-erythropoietic activity.

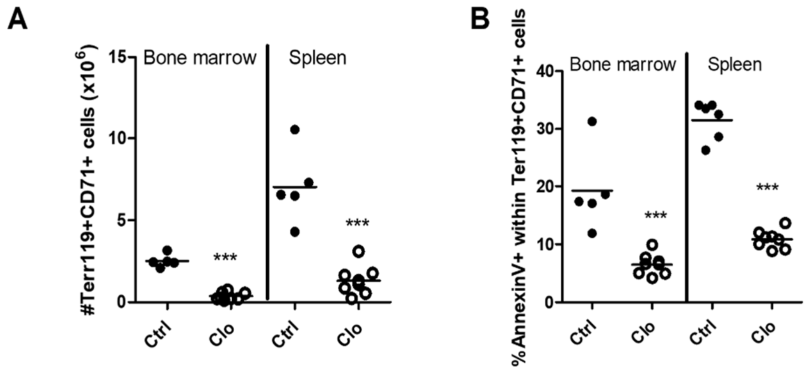

Figure 4. Assessment of total Ter119+ CD71+ cells and apoptosis levels in control mice and in mice treated with clodronate liposomes. (A) Compilation of Ter119+CD71+ cell numbers in the BM and spleen from control (Ctrl) and clodronate -treated (Clo) mice one week after injection of liposomes; significantly lower numbers of Ter119+ cells are found in mice ablated of macrophages. (B) The percentages of apoptotic annexin V+ Ter119+Cd71+ cells significantly decreased in the BM and spleen from clodronate treated mice. *** $p<0.001$.
